# Translation and linguistic validation of the Swedish recovering quality of life (ReQoL) – A brief research report

**DOI:** 10.3389/fpsyt.2023.1059406

**Published:** 2023-02-07

**Authors:** Elin Granholm Valmari, Marianne Melander, Gun-Marie Hariz, Matilda Naesström, Maria Lindström

**Affiliations:** ^1^Occupational Therapy Unit, Department of Community Medicine and Rehabilitation, Umeå University, Umeå, Sweden; ^2^Psychiatric Unit, Department of Clinical Sciences, Umeå University, Umeå, Sweden; ^3^Department of Clinical Science, Neurosciences, Umeå University, Umeå, Sweden

**Keywords:** mental illness, personal recovery, pilot testing, PROM, quality of life, QALY, linguistic validation, measure

## Abstract

In research and among clinicians, the focus has shifted from mainly symptom reduction and increasing functionality to a more recovery-oriented focus. Although there are instruments measuring recovery, there has been a lack of instruments sensitive enough to measure the quality of life for people with severe mental health disorders. Therefore, this study aimed to obtain a Swedish version of the Recovering Quality of Life (ReQoL) questionnaire adhering to best practice guidelines using various steps of translation, linguistic validation, and cognitive debriefing. The cognitive debriefing was conducted with seven participants, and all felt the items in the questionnaire were relevant to their health, apprehensible, and easy to complete. However, some issues were raised regarding wording and the concepts behind certain items. All feedback was considered, and some items were revised in response to criticism after continuous discussions. A Swedish version of ReQoL now exists, and although there is a need for ReQoL in different clinical research settings in Sweden, further research is required to psychometrically test the construct validity as well as reliability of the Swedish version in Sweden.

## 1. Introduction

Personal recovery has been defined as a profoundly personal and unique process for the individual to change their attitudes, values, feelings, goals, abilities, and roles to achieve a satisfactory, hopeful, and productive way of life despite the limitations of mental illness (1). Personal recovery differs from clinical recovery, which primarily centers on reducing symptoms and increasing functioning levels (2). Thus, researchers and clinicians have since the 1990s (3) shifted their attention from symptom reduction services to a more recovery-oriented praxis (1, 4). In later years, there has also been development toward a conceptual model for recovery based on personal recovery process frameworks and definitions including Connectedness, Hope and optimism about the future, Identity, Meaning in life, and Empowerment, abbreviated as CHIME. CHIME is today the most recognized existing framework for understanding personal recovery (5, 6), with the potential to enhance service users’ quality of life (7, 8).

This recovery movement includes a shift in focus away from instruments that only measure symptoms and toward more recovery-oriented factors (9). Although there are instruments measuring recovery (9, 10), there has been a lack of instruments measuring the quality of life for people with mental health disorders. This led to the development of a new patient-reported outcome measure (PROM) called the Recovering Quality of Life (ReQoL) by researchers at the University of Sheffield, England (11).

The ReQoL is based on CHIME and allows the calculation of quality-adjusted life years (QALYs) (11, 12). The need for such a PROM has been critical because assessing persons with severe mental illnesses, such as schizophrenia, has been difficult using more generic measures such as the EQ-5D-5L (focusing on pain and disability) or SF-36 (measuring pain and physical health) (13, 14). Both, to some extent, also evaluate general mental health, including depression and anxiety, but are not sensitive enough to capture symptoms and suffering at severe levels. Thus, this points to a need to expand the domains of quality of life for mental health since these can differ significantly between people with general mental illness and those with severe mental illness (15).

The ReQoL measures the recovering quality of life during the past week. It has two versions, a 10-item, and a 20-item version. The development process was conducted by a core team of researchers, supported by a scientific group, an international advisory group, a stakeholder group, and an expert service-user group (11), thus including public and patient involvement (16). The development of the ReQoL was done in five stages. The first and second stages included developing themes by conducting reviews and interviews with service users, after which a research team and service users conducted further item development. The last stages consisted of testing and analyzing the reliability, as well as the validity of the ReQoL with ongoing psychometric evaluations (11, 17, 18). ReQoL has also been translated into different languages and cross-culturally validated in over 20 countries (19–22).

In summary, mental health services in many countries have moved toward supporting personal recovery as a primary orientation (8), including Sweden, as demonstrated by Swedish national guidelines (23). Furthermore, healthcare management is organized to ensure high patient safety and good quality of care, including promoting cost efficiency (SFS 2017:30), stressing to care providers which target groups’ needs should be given priority, and how those needs should be met with good quality and cost-effective methods (24). As a result, calculating QALYs when recommending and carrying out interventions for people with mental illness is critical, as is using measures that are sensitive to cultural differences across countries.

Therefore, we translated the ReQoL into Swedish with permission from the ReQoL developers in Sheffield. The aim was to obtain a Swedish version of the ReQoL adhering to best practice guidelines, including translation, linguistic validation, and cognitive debriefing.

## 2. Methods

### 2.1. Translation, linguistic validation, and cognitive debriefing

The translation, linguistic validation, and cognitive debriefing followed the recommendations from the ReQoL developers, which are based on the ISPOR (International Society for Pharmacoeconomics and Outcomes Research) guidelines for cross-cultural validation of instruments (25). The Swedish translation is a fully validated version with a translation certificate from the distributor of the ReQoL instrument.

As a first step, the ReQoL developers provided a license to translate the ReQoL into Swedish in Sweden. To develop the Swedish version of the ReQoL, the quality of the translation was essential, with equivalence in terms and measured constructs. Therefore, an extensive translation procedure was followed according to the standard guidelines from the ReQoL developers. First, a dual forward translation from English into Swedish was undertaken by the last author and the fourth author. Then, a forward translation reconciliation was conducted by the second author. This task included finding a translation that most accurately represented the concepts within the source version and was also found culturally relevant and meaningful for use in Sweden.

The reconciled language version then underwent dual back translation from Swedish to English by two native English speakers, one of whom is an official translator. After this step, the first author conducted a back-translation review, where the back translations were compared to the source version to highlight any discrepancies in meaning or terminology. After this rigorous process, the Swedish version underwent a developer review, where the first author contacted the instrument developers and the developers reviewed the translation.

Then, the instrument went through cognitive debriefing according to the ReQoL recommendations of at least five native Swedish-speaking participants living in Sweden. We strived for recruitment in outpatient clinics and user organizations, where one participant had been educated in user-involved research. Thus, we conducted “cognitive debriefing” (25) with seven participants suffering from various mental or neuropsychiatric disorders, such as bipolar disease, attention-deficit/hyperactivity disorder, schizophrenia, depression, obsessive–compulsive disorder, and autism. Of the seven participants, four were men and three were women. Each patient was asked face-to-face questions while completing the PROM, providing feedback based on the experience of filling out the ReQoL questionnaire. They were urged to provide information regarding the questions, the response options, and the wording.

The last author conducted three interviews, the fourth author conducted two interviews, and the second author conducted two interviews. The first author then summarized the cognitive interviews and discussed them during meetings with the second author. Then the first and second authors recommended changes according to the participants’ feedback. Subsequently, final proofreading was conducted by a person not involved in the linguistic validation earlier (the third author) and the second author. After this step, the first and second authors had meetings to discuss specific wording and whether changes should be made to the PROM. The first and second authors also discussed discrepancies between the original ReQoL and this translation. Discrepancies in the interpretation or meaning of items led to some revisions of the Swedish translation. At the end of this stage, the Swedish ReQoL was sent back to the original developers for approval, some technicalities were adjusted, and then the translation was approved. A signed checklist of all steps of the translation process was sent back to the ReQoL distributors before the translation was officially finalized.

The Swedish Ethical Review Authority, Reg. No. 2020-06220, obtained ethical approval for this study as part of a larger research project, and all participants provided informed consent. A data management plan for the project can be found at: dmp.umu.se ID86475.

## 3. Results

During cognitive debriefing with the seven participants, they all felt that the questionnaire helped assess various areas relevant to their health and was easy to complete. They also found most of the items easy to understand and were able to complete the ReQoL questionnaire.

Three participants, however, had difficulty understanding the meaning of certain words or concepts underlying items, particularly when some words were more formally translated than words used in everyday life conversations. Their feedback was considered and discussed, thus changing the word “tasks” in item 1 (English version: I find it difficult to get started with everyday *tasks*) from the initially more formal Swedish translation “göromål” (tasks) to the colloquial word “sysslor” (chores). Also, in item 2 (English version: I felt *able* to trust others), the word “able” was changed from the initial Swedish translation “kapabel” (capable) to “hade förmåga att” (had the ability to).

Concerns were also raised about negations in item 6 (in English: I thought my life *was not worth living*), with regards to the meaning of the item as well as using negations in a sentence, which complicates scaling in Swedish. Thus, to avoid negations while still incorporating the correct meaning of the concept measured for that item, the word “värdelöst” (worthless) was used instead of the initial “har jag tänkt att mitt liv *inte var värt att leva*.” Concerns about Item 4 (in English: I could do the things I wanted to do) were raised regarding the meaning of having a life goal, but no changes were made to this item since only one participant reflected on it. Item 18 (in English: I had problems with my sleep) was also considered somewhat difficult to understand in terms of the question’s meaning and what it was intended to measure. However, the item was not changed, but the question was formatted into a different past tense form since some participants felt that the translation had grammatical inconsistencies regarding present and past tenses.

Regarding the scale, one participant noticed the difficulty in discriminating between the first, second, and third options in the response options (in English: 1st step, “Only occasionally,” 2nd step, “Sometimes,” 3rd step, “Often”). We had translated the first step to “Endast undantagsvis” (Only occasionally). Thus, the second step was changed to “Någon enstaka gång” (Once in a while), which separates the first, second, and third steps.

The main concerns expressed by the participants were the design and layout of the measure, which is standardized according to the ReQoL developers. This concern led to a discussion with the ReQoL developers about adding an explanation sentence in Swedish under their logo, as well as a suggestion to minimize text and numbers without importance for the target group, which could be a distraction while filling out the evaluation form.

## 4. Discussion and conclusion

The ReQoL questionnaire, developed in the United Kingdom and translated into over 20 languages (22) is now translated and linguistically validated into Swedish in Sweden, according to international best practice guidelines. The Swedish version of ReQoL can be found at Clinical Outcomes at Oxford University Innovation and their licensing portal: https://process.innovation.ox.ac.uk/clinical (22). So far, the translation process in Sweden has successfully achieved an acceptable and linguistically validated ReQoL questionnaire for Swedish-speaking persons with mental illness.

The translated and linguistically validated ReQoL can be used in different clinical as well as research settings in Sweden, including rehabilitation assessment. Translation of the ReQoL, for example, has been shown to be particularly applicable in a pragmatic cluster RCT led by the last author (ML) to evaluate the effect and cost-effectiveness of the Everyday Life Rehabilitation (ELR) model (26). ELR is an integrated, person-centered, activity- and recovery-oriented intervention in collaboration with and for persons with severe psychiatric disabilities living in sheltered or supported housing facilities (27). However, more psychometric testing is warranted to investigate reliability and construct validity in a Swedish setting to evaluate the PROM in different patient and user populations. Additionally, cultural validation for the translated Swedish version in other countries where Swedish is a common minority language, for example, Finland, should also be conducted, including psychometric testing.

In other countries, for example, in Singapore, psychometric performance using ReQoL within a first-episode psychosis intervention shows ReQoL to be suitable for routine use to measure recovering quality of life in psychiatric settings. ReQoL was also found to facilitate shared decision-making (28). In Germany, ReQoL was used to assess patients with major depression, dysthymia, and bipolar affective disorder, indicating that ReQoL is a promising measure for use in clinical settings and within research (29). Thus, other ReQoL translations have implicated both good construct validity and reliability in different countries (19, 20, 28, 29), in addition to the larger psychometric tests conducted in the British context (17, 30).

A couple of limitations should be addressed in this study. First, the cognitive debriefing only included seven participants, on whom we did not collect much demographic data on. To mitigate this, participants with different diagnoses and both sexes were included. A strength of the linguistic validation is also using standard guidelines from the ReQoL developers. The study’s lack of construct validity and reliability testing is another limitation. As a result, future validation work should be carried out to test the ReQoL’s psychometric properties in a Swedish context.

Concludingly, in this study, we have translated and linguistically validated the ReQoL questionnaire, a recovery measure where the quality of life is included with the possibility of conducting cost-effectiveness analyses by calculating QALYs. Although ReQoL has the potential to be used in other countries to assess the efficacy and cost-effectiveness of recovery-oriented interventions, more research in a Swedish context with different populations is needed, as is more psychometric testing.

## Data availability statement

The raw data supporting the conclusions of this article will be made available by the authors, without undue reservation.

## Ethics statement

The studies involving human participants were reviewed and approved by Swedish Ethical Review Authority, Reg. no. 2020-06220. The patients/participants provided their written informed consent to participate in this study.

## Author contributions

ML is the Principal Investigator (PI) for a larger project, including this study, its ethical considerations, a data-management plan, and the main responsibility for good clinical practice and methodology. EG and ML conceived the study. ML, MM, and MN conducted the cognitive debriefing. EG and MM were responsible for the first draft of the study, whereas G-MH, ML, and MN were responsible for writing specific sections and providing substantial feedback on the first draft. All authors have contributed by commenting, rewriting sections of the manuscript, and approving the final and submitted version of the manuscript.

## Funding

The funding for this study comes from the Department of Community Medicine and Rehabilitation, Umeå University, and partly from the Swedish Research Council for Health, Working Life, and Welfare (FORTE välfärdsforskning 2021-01391).

## Conflict of interest

The authors declare that the research was conducted in the absence of any commercial or financial relationships that could be construed as a potential conflict of interest.

## Publisher’s note

All claims expressed in this article are solely those of the authors and do not necessarily represent those of their affiliated organizations, or those of the publisher, the editors and the reviewers. Any product that may be evaluated in this article, or claim that may be made by its manufacturer, is not guaranteed or endorsed by the publisher.
